# Triploidy and Routine Combined First Trimester Pregnancy Screening

**Published:** 2019

**Authors:** Mitra Eftekhariyazdi, Ali Khaligh, Behnaz Suizi, Maryam Naghibi Nasab, Davood Zare-Abdollahi

**Affiliations:** 1. Department of Obstetrics and Gynecology, Faculty of Medicine, Sabzevar University of Medical Sciences, Sabzevar, Iran; 2. Sabzevar University of Medical Sciences, Sabzevar, Iran; 3. Genetics Research Center, University of Social Welfare and Rehabilitation Sciences, Tehran, Iran

**Keywords:** Fetal growth retardation, Hydrocephalus, Nuchal translucency measurement, Syndactyly, Triploidy

## Abstract

**Background:**

This report is about a pregnancy with a triploid fetus and underscores the potential of first trimester combined screening to detect this devastating chromosomal aberration earlier in pregnancy. This report is about a pregnancy with a triploid fetus identified from the first trimester combined screening and confirmed by amniocentesis.

**Methods:**

A 28 year old, G5P2AB2 woman was referred to our clinic at 15 weeks of gestation due to a remarkable decrease of her first trimester double biochemical markers and therefore in the high-risk range for trisomy 13 and 18. The woman underwent amniocentesis which revealed a karyotype of 69,XXX. The parents opted for termination and in post mortem physical examination, a hydrocephalus fetus with marked Intra-Uterine Growth Retardation (IUGR) in addition to syndactyly of third and fourth digits, low set malformed ears, micrognathia and club foot, was seen.

**Results:**

Our results and previous reports highlight the need to consider a somewhat consistent pattern of the first trimester combined screening in a pregnancy with triploidy and underscore the potential of this screening strategy to detect this chromosomal aberration earlier in pregnancy.

**Conclusion:**

Early prenatal diagnosis of this syndrome would provide women an opportunity to terminate an affected pregnancy earlier. This is also important in preventing the risks of associated later induced abortion or obstetric complications.

## Introduction

Several studies have investigated the efficacy of the first trimester combined screening and insist on its acceptable detection rate of common aneuploidies (*i.e*. 13, 18, and 21 trisomies) 1,2. It is well established that trisomy 21 pregnancies are associated with increased maternal age, increased fetal Nuchal Translucency (NT) thickness, increased maternal serum free Beta Human Chorionic Gonadotropin (free BHCG) and decreased maternal serum Pregnancy Associated Plasma Protein-A (PAPP-A) 3. Applying this strategy, several studies reported that about 85–90% of affected pregnancies, for a screen positive rate of 5%, are identified 4. Trisomies 13 and 18 are characterized by increased fetal NT and decreased maternal serum free BHCG and PAPP-A. Again, combined screening can identify 85–90% of affected pregnancies for a 0.5±1.0% and 0.1± 0.5% false positive rate, respectively 5. For the Turner syndrome, although there is not a consistent and reproducible pattern, increased NT, normal maternal serum free BHCG and low PAPP-A is the accepted recurrent pattern that is able to identify majority of the cases, over 90% 1,2.

Beside this unprecedented high detection rate of common aneuploidies, some studies highlighted the usefulness of this screening strategy to detect a much broader range of chromosomal aberrations, including triploidy, small supernumerary marker chromosomes and rare numerical and/or structural chromosomal abnormalities 5. It is estimated that triploidy occurs in 1% of all conceptions but most of the affected fetus cannot survive the first trimester period and the majority of these pregnancies abort spontaneously 6. Rarely, such pregnancies can progress and survive the second and third trimesters. The prevalence of triploidy at the 11th to 14th week scan and in newborns is around 1:3300 and 1:10000, respectively, highlighting a strong negative selection on triploid pregnancies 2,6,7. Triploidy can be classified into two categories depending on the parental origin of the extra chromosome set and placental and/or ultrasound findings. In type I, the additional chromosome set is of paternal origin (Diandric) and placenta is enlarged and partially multicystic (molar) whereas the fetus is relatively well-grown with either proportionate head size or slight microcephaly. Type II, which is the most common, extra set of the chromosome is of maternal origin (digynic) and is characterized by a small normal looking placenta and severely growth restricted fetus with pronounced wasting of the body and sparing of the head 2. Regarding the first trimester combined screening, although the fetal NT in triploidy does not markedly deviate from that of euploid fetuses, the biochemical markers show large deviations. Diandric triploidy is characterized by a combination of increased free BHCGMoM and moderately decreased PAPP-A MoM, similar to the biochemical profile of cases of trisomy 21. On the other hand, in cases of digynic triploidy, the biochemistry demonstrates markedly decreased free BHCG and PAPP-A MoMs^2^,^8^.

This report is about a pregnancy with a triploid fetus and underscores the potential of first trimester combined screening to detect this devastating chromosomal aberration earlier in pregnancy.

## Patients and Methods

**Figure 1.**
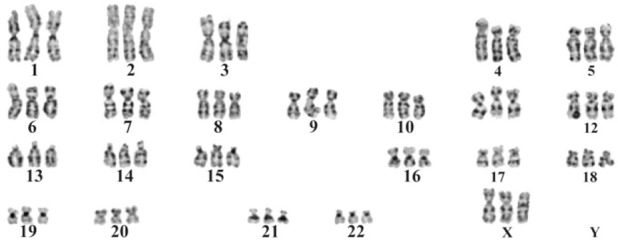
Karyotype result showing 69,XXX.

**Figure 2.**
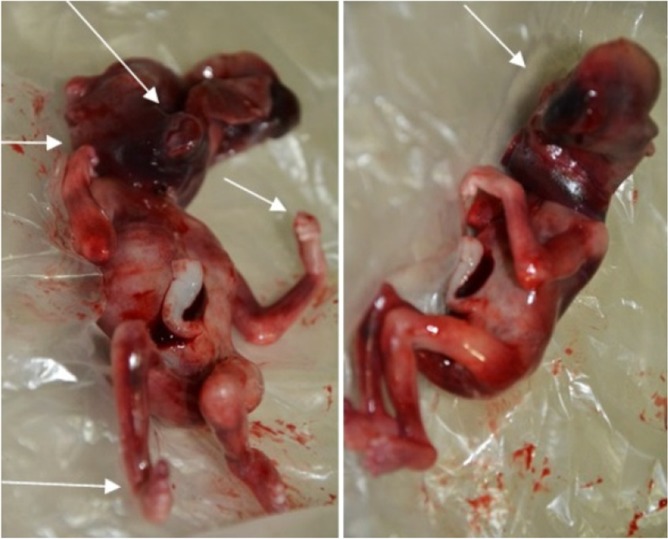
Images of triploid fetus. Arrows show the phenotypes highly associated with triploidy including hydrocephaly, low set ears, bilateral syndactyly of the third and fourth digits and club foot.

A 28 year old, G5P2AB2 woman was admitted to our clinic (Shahidan Mobini Hospital, Sabzevar University of Medical Sciences, Sabzevar, Khorasan Razavi, Iran) for perinatologist counseling at 15 weeks of gestation due to remarkable decrease of her first trimester double biochemical markers and therefore in the high-risk range for trisomy 13 and 18. Her first trimester combined screening included NT=1.8 *mm* (CRL=58, 1.55 *MoM*) and remarkable reduced PAPP-A and free BHCG (0.06 *MoM* and 0.13 *MoM*, respectively). Amniocentesis was highly recommended according to the approved national pregnancy screening program. The woman underwent amniocentesis which in primarily quantitative fluorescent polymerase chain reaction (QF-PCR), the patterns of the markers were consistent with a triploid fetus and confirmed by the cultured amniocytes, revealing a karyotype of 69, XXX (Figure 1). After genetic counseling, the parents opted for pregnancy termination. Written informed consent was obtained from parents and in physical examination, a hydrocephalus fetus with marked Intra-Uterine Growth Retardation (IUGR) in addition to syndactyly of third and fourth digits, low set malformed ears, micrognathia and club foot was seen (Figure 2).

## Discussion

Among chromosomal disorders, triploidies are the most commonly observed chromosomal aberration at conception and its frequency may be as high as 1:100 9. However, the majority of these triploid cases abort during the first trimester or even later as spontaneous abortions, so that ultimately it leads to the rarity of the triploidy in cases that underwent amniocentesis, approximately 1:3300 9. Eventually, the incidence rate reaches the lowest in live-born, approximately 1:10,000 10. Regardless of the lethality of the triploidy as a general rule, as shown in our case, some triploid pregnancies can continue until the later stages of pregnancy and impose great concern to the families. Routine first trimester screening strategy has the potential to detect a much broader range of chromosomal aberrations, including triploidy, small supernumerary marker chromosomes and rare numerical and/or structural chromosomal abnormalities in addition to common aneuploidies (*i.e*. 13, 18 and 21). Given a triploid pregnancy, regardless of the NT value, double marker MoM to a large degree shows a characteristic pattern. In that, in diandric triploidy, a combination of increased free BHCG MoM and moderately decreased PAPP-A MoM can be seen. On the other hand, in cases of digynic triploidy, double markers demonstrate markedly decreased free BHCG and PAPP-A MoMs 2,8. In our report, the potential of this biochemical combination was emphasized to predict and/or confirm a triploid pregnancy in earlier stages of pregnancy or in confronting with a triploid karyotype result after amniocentesis.

Taking in to account the routine screening strategies, using combined, quadruple marker and sequential strategies, the detection rate of the triploid pregnancies was defined as 85, 78.1 and 91%, respectively 1–3. These findings assert that triploidy is a disorder that can incidentally be detected as a part of a screening program for common aneuploidies and/or NTDs and high light the usefulness and high detection rate of triploidy using these screening strategies.

Many malformations have been reported in association with triploidy including hydrocephaly and syndictyly, especially of the third and fourth fingers, low set ears, cleft lip/palate, clubfeet which are present in about two third of the triploid fetuses in addition to the remarkable IUGR, which does not affect the head as severely as the body. These malformation collectively led to the distinctive feature of the triploidy 11. Holoprosencephaly, encephalocele, myelomeningocele, dysplastic calvaria, agenesis of the corpus callosum, ventriculomegaly, Arnold-Chiari malformation, omphalocele, gastroschisis, low nasal bridge, exophthalmia, heart defects, and renal agenesis are the other described associated abnormalities 7,11.

In post mortem physical examination of the present case, a hydrocephalus fetus with IUGR, low set ears and limb abnormalities including bilateral syndactyly of the third and fourth digits and club foot was observed (Figure 2).

Although, as a shortcoming, information on parental origins of the extra chromosome set was not available in this study, based on the available literature, more cases with the serum marker patterns of digynic were seen in amniocentesis stage than diandric ones 2. Based on this premise and normal looking placenta in our case, it seems that extra chromosome set is of maternal origin, although this should be verified through Short Tandem Repeats (STRs) study.

## Conclusion

Overall, although there is questionable advantage in screening for triploidy from the view of birth defect prevention, early prediction and diagnosis of this syndrome would provide women an opportunity to terminate an affected pregnancy sooner, which might have a high risk of associated obstetric complications. The results of this study also provide useful information for prenatal genetic counseling.
